# Single-tablet regimens (STRs) enhance patients’ acceptability of HAART

**DOI:** 10.7448/IAS.15.6.18103

**Published:** 2012-11-11

**Authors:** F Maggiolo, S Fregosi, P Bortolussi, S Marcotullio, R Murri

**Affiliations:** 1 Ospedali Riuniti, Department of Infectious Diseases, Bergamo, Italy; 2 GfK Eurisko, Healthcare Department, Milan, Italy; 3 Nadir Onlus Foundation, Rome, Italy; 4 Catholic University, Department of Infectious Diseases, Rome, Italy

## Abstract

Patients’ acceptability of HAART is a subjective variable that may deeply influence therapeutic outcome. The feeling of the patient may alter adherence and lead to virologic failure. Acceptability may depend on various variables often difficulty evaluated by the care-giver. In a clinical setting the evaluation of acceptability is difficult, too, as patients may feel a judgement and be less sincere. Aim of this study was to asses adherence and acceptability of HAART. To limit reporting biases, the study was performed in five different non-clinic settings covering North and Central Italy. A total of 230 patients on stable HAART were asked to complete a semi-structured, anonymous questionnaire reporting their attitude toward HAART, their adherence and the acceptability of their regimen. In these notes we focus on this last patient-oriented outcome. Most of the subjects were males (66%) with a mean age of 46 years, with higher education level (72%) and a long history of HIV infection (mean 13.6 years). Consequently only 17% of patients were on a first-line regimen. Patients reporting a high or very high acceptability of HAART were 60% compared to a 31% reporting a fair grade of satisfaction and a 9% indicating low or null acceptability. However the type of the regimen significantly influenced patients’ acceptability. Single-tablet regimens (STRs), OD regimens with more than one tablet/day or BID regimens were scored as highly acceptable in 84%; 61%; and 53% of cases, respectively (P < 0.0001) (Figure). Statistical significance was retained when the dosing schedule was entered in a multivariate logistic model. When the analysis was restricted to experienced patients 62% of them were currently on a regimen based on a reduced number of pills compared to the previous one. Patients scored the previous regimen as more difficult to comply with in 72% of cases; as difficult in 22% and less difficult in 6%. The eventuality of AEs (40%); respect of timing of pill intake (39%) and number of pills (27%) were the major reasons of patients’ low acceptability of HAART. High acceptability is one of the winning characteristics of a regimen, favoring long-term adherence, durability and efficacy. Although highly subjective, acceptability may be positively influenced by characteristics of the HAART regimen such as simplicity. According to our results, STRs show a higher acceptability compared to more complex regimens.

**Figure 1:**
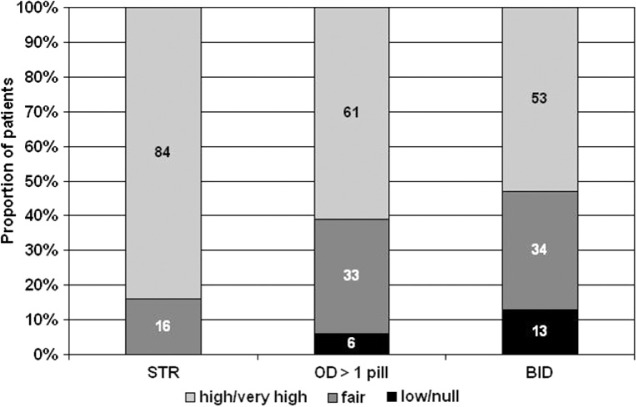
Patient reported acceptability for thier current HAART regimen.

Patient reported acceptability for thier current HAART regimen.

